# Decreased Resting-State Connectivity between Neurocognitive Networks in Treatment Resistant Depression

**DOI:** 10.3389/fpsyt.2015.00028

**Published:** 2015-03-02

**Authors:** Bart P. de Kwaasteniet, Maria M. Rive, Henricus G. Ruhé, Aart H. Schene, Dick J. Veltman, Lisanne Fellinger, Guido A. van Wingen, Damiaan Denys

**Affiliations:** ^1^Department of Psychiatry, Academic Medical Center, Amsterdam, Netherlands; ^2^Brain Imaging Center, Academic Medical Center, Amsterdam, Netherlands; ^3^Department of Psychiatry, Mood and Anxiety Disorders, University Medical Center Groningen, University of Groningen, Groningen, Netherlands; ^4^Department of Psychiatry, Radboud University Medical Center, Nijmegen, Netherlands; ^5^Donders Institute for Brain, Cognition and Behavior, Radboud University Nijmegen, Nijmegen, Netherlands; ^6^Department of Psychiatry, VU University Medical Center, Amsterdam, Netherlands; ^7^Netherlands Institute for Neuroscience, Royal Netherlands Academy of Arts and Sciences, Amsterdam, Netherlands

**Keywords:** major depressive disorder, treatment resistant depression, functional connectivity, salience network, cognitive control network, default mode network

## Abstract

Approximately one-third of patients with major depressive disorder (MDD) do not achieve remission after various treatment options and develop treatment resistant depression (TRD). So far, little is known about the pathophysiology of TRD. Studies in MDD patients showed aberrant functional connectivity (FC) of three “core” neurocognitive networks: the salience network (SN), cognitive control network (CCN), and default mode network (DMN). We used a cross-sectional design and performed resting-state FC MRI to assess connectivity of the SN, CCN, and both anterior and posterior DMN in 17 severe TRD, 18 non-TRD, and 18 healthy control (HC) subjects. Relative to both non-TRD and HC subjects, TRD patients showed decreased FC between the dorsolateral prefrontal cortex and angular gyrus, which suggests reduced FC between the CCN and DMN, and reduced FC between the medial prefrontal cortex and precuneus/cuneus, which suggests reduced FC between the anterior and posterior DMN. No significant differences in SN FC were observed. Our results suggest that TRD is characterized by a disturbance in neurocognitive networks relative to non-TRD and HC.

## Introduction

About one-third of patients with major depressive disorder (MDD) do not respond to two or more adequate prescribed antidepressants. They are considered to be suffering from treatment resistant depression (TRD) (1, 2), which is associated with an overall worse prognosis and higher medical costs (3). Only few neuroimaging studies have investigated the neural mechanisms underlying TRD, mostly focusing on regional brain activity and yielding equivocal results (4, 5). The investigation of network dysfunction in TRD can be guided by its investigation in the larger group of MDD, which has been studied more extensively. MDD has typically been associated with functional brain abnormalities including aberrant functional connectivity (FC) between distant brain areas (6–9). FC analysis quantifies the temporal correlation of neuronal activity patterns of anatomically separated brain regions (10, 11) and can be used to investigate the interaction within and between brain networks. Several studies showed aberrant FC within and between the salience network (SN) (12, 13), cognitive control network (CCN) (12), and default mode network (DMN) (7, 8, 12) in depressive patients.

The SN comprises the anterior insular cortex and dorsal anterior cingulate cortex (ACC), which serves to evaluate the relevance of internal and external stimuli in order to generate appropriate responses and guide behavior (14). The anterior insula within the SN is critically involved in maintaining and updating representations of current and predictive salience (15). Particularly, the right anterior insula has been suggested to critically contribute to appropriate behavioral responses to salient stimuli via switching between DMN-related self-referential and CCN-related goal directed cognitive activity (16). The CCN includes the dorsolateral prefrontal cortex (DLPFC) and pregenual ACC (17, 18), which is associated with top-down modulation of attention and regulation of the affective response (19, 20). The DMN comprises both the posterior cingulate cortex (PCC) and medial prefrontal cortex (MPFC) as core areas (21, 22) and the precuneus and temporo-parietal cortices. The DMN underlies the mental process of introspection – the mind turning inward as it moves away from externally focused thoughts (23–25). One study investigating the FC between these networks showed decreased FC between the DMN and CCN as well as increased connectivity between the SN and DMN in MDD relative to healthy controls (HC) (12). However, another independent component analysis (ICA) study in adolescent depression, could not replicate this abnormal connectivity between the DMN and CCN (26). Furthermore, within the SN decreased FC was found in the left and right anterior insula, as well as increased FC in the left and right ACC in MDD patients, and, within the DMN, increased FC in the precuneus (12). Other studies investigating DLPFC FC showed increased connectivity of anterior regions of the DMN (including the MPFC and ACC) in MDD patients (27, 28). Within posterior regions of the DMN (including the PCC and precuneus) increased connectivity was demonstrated (8, 27, 29), but decreased connectivity of posterior DMN regions has also been reported (28). Furthermore, a granger causality analysis showed decreased connectivity between anterior and posterior DMN regions in MDD patients relative to HC (29).

Regarding TRD, limited knowledge of these networks and their function exists. Two FDG-PET studies showed decreased metabolism of prefrontal and DMN areas in TRD patients relative to non-TRD patients including the DLPFC, dorsal ACC, premotor cortex, and insula. In contrast, higher metabolism in the uncus, left amygdala, and subgenual ACC were found (30, 31). In TRD relative to HC, Mayberg et al. showed a similar pattern of increased metabolism of the subgenual ACC and decreased metabolism of the DLPFC, dorsal ACC, premotor cortex, and insula (30). Based on these findings, a neurobiological model can be defined, in which both TRD and non-TRD are characterized by DMN, CCN, and SN abnormalities. Hypothetically, these abnormalities are more profound in TRD compared to non-TRD patients, given the fact that TRD subjects are more severely affected. One resting-state fMRI study investigated FC between resting-state networks in TRD, and showed decreased connectivity in several networks including the SN, CCN, and DMN in comparison to HC (32). This pattern was similar to that of non-TRD patients, though a direct comparison between TRD and non-TRD showed relative sparing of the SN in TRD. However, this study included patients with relatively mild TRD, i.e., patients not responding to two different modern antidepressants, which may explain why there were little connectivity differences between the TRD and non-TRD group. Although non-response to two different modern antidepressants is the formal definition of TRD (33), patients may subsequently respond to treatment with an irreversible monoamine oxidase (MAO) inhibitor or after electroconvulsive therapy (ECT). Patients who do not respond to all those forms of treatment can be considered more severely treatment resistant, but even less is known about the pathophysiology of this specific group of patients.

In the present study, we used a cross-sectional design to investigate FC of the three neurocognitive networks (SN, CCN, and DMN) in a group of severe TRD patients who had not responded to at least four different antidepressants and ≥6 sessions of bilateral ECT (given at least 3 months before scanning). We hypothesized that severe TRD would be associated with reduced FC in comparison to non-TRD patients and HC.

## Materials and Methods

### Subjects

TRD and non-TRD patients were recruited at the Departments of Psychiatry of the Academic Medical Center (AMC) in Amsterdam and St. Elisabeth Hospital in Tilburg, The Netherlands. HC were recruited via advertisements. The study was approved by the Medical Ethical Committees of both hospitals and all subjects provided written informed consent. The TRD and non-TRD patients originally participated in a study on deep brain stimulation (DBS study) and one on recurrent depression (DIADE study), respectively. General inclusion criteria in both studies, and hence for both MDD groups were (i) age between 18 and 65 years; (ii) total score on Hamilton depression rating scale (HAM-D) ≥18; (iii) primary diagnosis of MDD according to the DSM-IV criteria and assessed by the structured clinical interview for DSM-IV disorders (SCID) (34). In the DBS study, the most severely treatment resistant patients were selected and were only included with an illness duration of >2 years, and not responding to (i) at least two adequate treatments of two different modern antidepressants (SSRI, SNRI, or NaSSA), (ii) a tricyclic antidepressant, (iii) an irreversible MAO inhibitor, and (iv) at least six sessions of bilateral ECT. In the DIADE study, only medication-free patients with a history of at least two major depressive episodes with remission in between (either spontaneously or with treatment) were included; these patients were considered as non-TRD. Furthermore, patients from the DIADE study were excluded when they did not achieve remission during a follow-up period of 2.5 years after scanning. Exclusion criteria in both studies were (i) Parkinson’s disease, dementia, or epilepsy; (ii) bipolar disorder; (iii) schizophrenia or a history of psychosis unrelated to MDD; (iv) alcohol or substance abuse during last 6 months; and (v) antisocial personality disorder. HC were screened by the SCID. None of the healthy participants reported a family history of psychiatric illness. Half of the HC were derived from the DBS study, and the other half from the DIADE study.

The HAM-D scale (35) was used to quantify depression severity, the International Standard Classification of Education 1997 (ISCED-1997) (36) to classify education level and the Maudsley staging method (MSM) to quantify the level of treatment resistance (33, 37). The MSM score includes various clinical parameters: duration of the current depressive episode, symptom severity, and level of functioning as measured by the global assessment of functioning score (GAF). For a complete list of these clinical variables, we refer to Fekadu et al. (37).

### Study design

We used a cross-sectional study design in which patients from both MDD groups were scanned during their current depressive episode at the moment they met the inclusion criteria.

### MRI data acquisition

All resting-state and structural MRI data were acquired on a 3.0 T MRI scanner (Philips Intera, Philips Medical Systems, Best, The Netherlands) in the AMC, with body coil excitation and an eight-channel SENSE head coil. The head was held in place with a headphone and foam-pads. For the functional resting-state scan, the following parameters were used: echo time 30 ms, repetition time 2300 ms, flip angle 80°, matrix 96 × 96, number of slices 35, slice gap 0 mm, slice thickness 3 mm, ascending slice order, field of view 220 mm × 220 mm, voxel size 3 mm × 2.29 mm × 2.29 mm, SENSE factor 2. In total 200 volumes were acquired with a total duration of 7 min and 51 s. The resting-state fMRI scans of the non-TRD group and nine HC subjects were acquired with identical parameters except for the number of slices (38), leading to differences in brain coverage. To ensure identical brain coverage for data analysis, an inclusive mask was used that only included data that were present in all subjects. For anatomical co-registration, a 7-min T1-weighted structural image was acquired. TRD patients and one half of controls had their eyes open during scanning whereas the non-TRD patients and the other half of controls had their eyes closed.

### fMRI data analysis

Statistical Parametric Mapping (SPM8, Wellcome Trust Centre for Neuroimaging, London, UK)^1^ was used for the following preprocessing steps: realignment to correct for subject motion; slice timing; co-registration of functional and structural data; spatial normalization into standard stereotactic space using a template from the Montreal Neurological Institute (MNI) and resampling to 4 mm isotropic voxels; smoothing of data with an 8 mm Gaussian kernel. The realignment parameters were inspected to ensure minimal head movement during scanning. Movement was limited to <3 mm in any direction (1 slice thickness), so all data could be used for analysis.

### Functional connectivity analysis

We performed seed region FC analyses to investigate network connectivity. The resting-state fMRI data analysis toolkit (REST) software package^2^ was used for the FC analysis. For each network we selected one seed region (Figure S1 in Supplementary Material). Within the SN, we selected the anterior insula, which is a core area within this network (15, 38). It was defined as two separate spherical seed ROIs (radius = 4 mm) for the left (−32, 24, −6 mm) and right anterior insula (37, 25, −4 mm) on the basis of a previous study, showing that this region initiates switching between the CCN and DMN (16). Within the CCN, we selected the DLPFC as seed region, which has consistently shown decreased task-related activity in MDD (23, 25). In line with a key resting-state FC study in MDD, we defined two separate spherical seed regions (radius = 4 mm) for the left and right DLPFC (±36, 27, 29 mm) (9). Within the DMN both the PCC and MPFC were selected as seed regions since they are core areas within posterior and anterior parts of this network (21, 22). Following the resting-state studies by Fox et al. (22) and Biswal et al. (39), we defined the MPFC as a sphere (radius = 4 mm) around coordinate (−1, 47, −4 mm) based on a previous meta-analysis of network activation during rest (40). For the definition of the PCC, we used the peak coordinates (−2, −51, 27 mm with radius = 4 mm) from a study that showed that the DMN is composed of distinct subunits (41).

Using REST, the linear trend of the MRI time series of each subject was removed and data were filtered with a bandpass filter between 0.01 and 0.08 Hz. Next, we derived estimates of white matter and cerebrospinal fluid fluctuations as well as head movement to include in the regression analyses. Voxel-wise correlation analyses were performed between each seed region and the rest of the brain. The correlation coefficients in each voxel were transformed to *Z*-scores using the Fisher *r*-to-*z* transformation to adjust the variance of correlation coefficients for group level comparisons. Fisher’s *Z*-scores of each subject were entered in a second level analysis to determine the differences in FC between the TRD, non-TRD, and control group within a study-specific brain mask. All statistical tests were family wise error (FWE) rate corrected (*p* < 0.05) for multiple comparisons at the cluster level (using a cluster-forming threshold of *p* < 0.005 uncorrected) across the entire brain. We first tested the main effect of group with an exploratory *F*-test using the non-stationary toolbox for SPM to acquire results on the cluster level with stationarity assumption^3^. Then for each significant cluster *post hoc T*-tests were performed to investigate differences between the following groups: TRD versus non-TRD, TRD versus HC, and non-TRD versus HC. In addition to these primary analyses, we investigated the potential effect of age and gender by including these variables as covariates of no interest in the analysis.

### Reference functional connectivity analyses

To investigate the specificity of the FC analyses, we additionally performed a reference connectivity analysis with the left and right primary motor cortex using similar methodology. Coordinates from Geyer et al. (42) were used to define spherical (radius = 4 mm) seed regions for left (−22, −30, 64) and right (21, −30, 65) motor cortex. In addition, to ensure that the connectivity analyses from the SN, CCN, and DMN, as well as the motor cortex connectivity analysis could not be explained by the methodological differences, we performed two additional connectivity analyses: (I) comparison between the TRD group and those HC who were investigated with identical scanning protocol and instructions; and (II) a connectivity analysis from a white matter seed region. To this end, a spherical seed region (−20, 20, 26 mm) with a radius of 2 mm was defined by use of the WFU-Pickatlas (43, 44).

## Results

### Patient characteristics

We included 17 TRD patients, 18 non-TRD patients, and 18 HC subjects (Table 1). The TRD, non-TRD, and HC group were not significantly different regarding age (*p* = 0.40), sex (*p* = 0.68), and education level (*p* = 0.71). The mean ages of these groups were 52.5 (±9.3), 48.9 (±7.3), and 51.5 (±7.6), respectively. HAM-D scores were not significantly different (*p* = 0.61) between the TRD and non-TRD group; mean HAM-D for the TRD group was 21.8 (±4.9) and 20.9 (±5.2) for the non-TRD group (corresponding with moderate–severe depression). All TRD patients used different psychotropic drugs at time of scanning whereas all non-TRD patients were free of medication during scanning. Mean MSM scores were 12.0 (±1.8) for the TRD and 5.6 (±1.7) for the non-TRD group, which – as expected – indicates a more severe level of treatment resistance in the TRD group and a mild level of treatment resistance in the non-TRD group (*p* < 0.01). Furthermore, the age of onset was not significantly different (*p* = 0.30) between the TRD (32.2 ± 13.9 years) and non-TRD group (28.2 ± 8.3 years). By design, the duration of the current depressive episode and the number of past treatments were significantly higher in the TRD group (both *p* < 0.01): 83.3 (±44.6) months and 6.5 (±2.7) treatments in TRD versus 11.9 (±8.9) months and 1.8 (±2.6) treatments in MDD.

**Table 1:** **Demographic and clinical characteristics of TRD, non-TRD, and healthy controls subjects**.

	TRD (*n* = 17)	Non-TRD (*n* = 18)	Healthy controls (*n* = 18)	Between group comparison (*p*-values)
Age (SD)	52.5 (9.3)	48.9 (7.3)	51.5 (7.6)	0.40^a^
Gender (m/f)	8/9	6/12	8/10	0.68^b^
HAM-D (SD)	21.8 (4.9)	20.9 (5.2)	n.a.	0.61^a^
Education level (median/IQR)	4/3	4/3	4/2	0.71^c^
MSM score (SD)	12 (1.8)	5.6 (1.7)	n.a.	<0.01^a^
Medication use	17/17^d^	0/18	n.a.	<0.01^b^
Duration of current episode in months (SD)	83.3 (44.6)	11.9 (8.9)	n.a.	<0.01^a^
Age of onset (SD)	32.2 (13.9)	28.2 (8.3)	n.a.	0.30^a^
Number of past antidepressant treatments	6.5 (2.7)	1.8 (2.6)^e^	n.a.	<0.01^a^
Psychotherapy (yes/no)	15/2	18/0	n.a.	<0.01^b^

*TRD, treatment resistant depression; non-TRD, major depressive disorder subsequently responsive to treatment (see Materials and Methods); HAM-D, Hamilton depression rating scale; ISCED, international standard classification of education; IQR, interquartile range; SD, standard deviation; m/f, male/female; MSM, Maudsley staging method*.

*^a^One-way ANOVA*.

*^b^Chi-square test*.

*^c^Kruskal–Wallis test*.

*^d^Use of antidepressants at time of scanning in TRD patients: selective serotonin reuptake inhibitor (*n* = 4), selective noradrenalin reuptake inhibitor (*n* = 2), tricyclic antidepressant (*n* = 2), monoamine oxidase inhibitor (*n* = 2), and typical or atypical antipsychotics (*n* = 10)*.

*^e^Two missing values in the non-TRD group*.

### Resting-state networks

**Figure 1:**
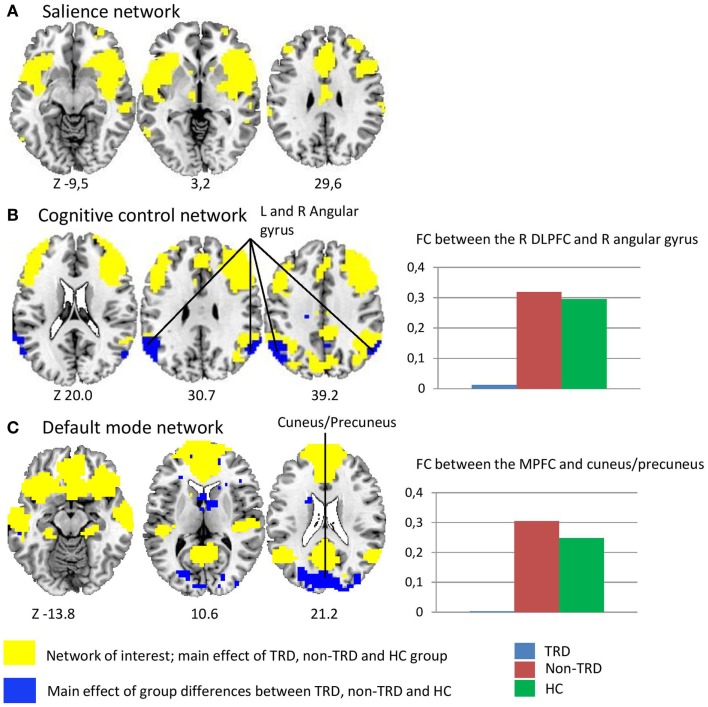
**Differences in resting-state functional connectivity between treatment resistant depression, non-treatment resistant depression, and healthy controls**. The yellow areas represent the networks of interest, which were defined by whole-brain voxel-wise correlations with the three different seed ROIs across groups: (A) the salience network (SN) consisted of the anterior insula, dorsal ACC, and ventrolateral prefrontal cortex (B) the cognitive control network (CCN) consisted of the DLPFC, dorsal ACC, and inferior parietal gyrus (C) the posterior and anterior default mode network (DMN) consisted of overlapping regions including the PCC, precuneus, medial prefrontal cortex, middle temporal gyrus, and parahippocampal gyrus. The blue areas represent the main effect of group, showing differences between the TRD, non-TRD, and healthy control group. **(A)** *SN*; the main effect of group shows no between group differences between the SN and both the CCN and DMN. **(B)** *CCN*; the main effect of group shows decreased functional connectivity between the right DLPFC and left angular gyrus between the three groups suggesting decreased connectivity between the CCN and the DMN. The spectrum plot visualizes specific decreases in connectivity in TRD patients relative to both non-TRD patients and healthy controls. **(C)** *DMN*; the main effect of group shows decreased functional connectivity between the MPFC and the cuneus/precuneus between the three groups suggesting decreased connectivity between the anterior and posterior DMN. The spectrum plot again visualizes a specific decreased functional connectivity in TRD patients relative to both non-TRD patients and healthy controls. The panels illustrate the significant clusters at *p* < 0.05 FWE corrected. The *Z* coordinates of the transversal planes are in Montreal Neurological Institute (MNI) space and the yellow areas are overlaid by the blue areas.

To probe the networks of interest, we assessed whole-brain voxel-wise positive correlations with the three seed ROIs (*p* < 0.05 FWE corrected, Figure 1). These analyses showed that the CCN consisted of the DLPFC, dorsal ACC, caudate, and inferior parietal gyrus. The posterior and anterior DMN consisted of largely overlapping regions including the PCC, precuneus, angular gyrus, MPFC, and parahippocampal gyrus. The SN consisted of bilateral anterior insula cortex, ventrolateral prefrontal cortex (VLPFC), and dorsal ACC.

### Functional connectivity analysis

For the CNN with the right DLPFC seed, the main effect of group showed a significant cluster in the left angular gyrus (*p* = 0.002). In this cluster, *post hoc* tests showed decreased FC in TRD relative to non-TRD (*p* = 0.003) as well as HC subjects, whereas no significant differences between non-TRD patients and HC were observed. These results therefore suggest decreased connectivity between the CCN and the DMN, specifically in TRD patients (Figure 1; Table 1). Regarding the anterior DMN with the MPFC seed, the main effect of group showed a significant cluster in the cuneus/precuneus (*p* < 0.001). Within this cluster, *post hoc* tests showed decreased connectivity in TRD relative to both non-TRD (*p* < 0.001) and HC subjects whereas the comparison between non-TRD and HC subjects did not show significant differences. These results suggest decreased connectivity between the anterior and posterior parts of the DMN, again specifically in TRD patients (Figure 1; Table 2). The main effect of group showed no significant differences in SN connectivity. Correcting for age and gender did not alter this pattern of results.

**Table 2:** **Functional connectivity analyses in comparison between TRD, non-TRD, and healthy controls**.

Comparison	Brain region	MNI coordinates			Cluster size	p*
		*x*	*y*	*z*		
**SEED REGION: RIGHT DLPFC**						
Main effect of group	Left angular gyrus	−58	−56	34	141	0.002
Non-TRD > TRD	Left angular gyrus	−58	−56	34	189	0.003
	Right angular gyrus	62	−60	30	118	0.029
Non-TRD > HC	None					
HC > TRD	Left angular gyrus	−58	−52	30	213	0.002
**SEED REGION: MPFC**						
Main effect of group	Cuneus/precuneus	2	−84	30	365	<0.001
Non-TRD > TRD	Cuneus/precuneus	10	−88	26	766	<0.001
	Left superior parietal gyrus	−14	−48	78	160	0.005
Non-TRD > HC	None					
HC > TRD	Cuneus/precuneus	−6	−92	22	281	<0.001

*HC, healthy controls; TRD, treatment resistant depression; non-TRD, non-treatment resistant depression; MNI, Montreal Neurological Institute space coordinates*.

*Corrected for multiple comparisons with family wise error correction (FWE) on cluster level.

To further ensure that the results could not be explained by methodological differences (eyes open/closed and the difference in number of slices), we compared the TRD group with a smaller number of HC who were investigated with identical scanning protocol and instructions. Similar to the results of the TRD versus all HC comparison, this TRD versus HC subgroup comparison showed decreased FC in the TRD patients between the right DLPFC and left angular gyrus (*p* = 0.007, FWE corrected at cluster level). The results from the MPFC seed region also showed a similar pattern of decreased connectivity with the cuneus/precuneus in TRD patients relative to these HC (*p* < 0.005 uncorrected, *k* = 21), but failed to reach significance when FWE correction for multiple comparisons was applied (*p* = 0.854). Nevertheless, these findings suggest that the methodological differences between the non-TRD and TRD group had little influence on the present results.

### Reference functional connectivity analyses

We probed FC from the left and right primary motor cortex to assess whether the connectivity differences generalized beyond the neurocognitive networks. We observed a similar pattern of reduced FC in TRD patients relative to both non-TRD patients and HC between the right motor cortex and right superior temporal gyrus as well as between the left motor cortex and the left superior temporal gyrus. This suggests that TRD pathophysiology is not limited to abnormalities between the neurocognitive networks but is associated with a more widespread pattern of decreased resting-state connectivity (Table S1 in Supplementary Material). To ensure that the consistent pattern of reduced connectivity in TRD was not caused by signal fluctuations that are of non-neuronal origin, we performed an additional control analysis with a white matter seed, even though the analyses already accounted for motion, white matter, and cerebral spinal fluid fluctuations. The main effect of group of the white matter FC analysis showed no significant differences between the TRD, non-TRD, and HC group. This suggests that the network connectivity analyses from the three neurocognitive networks, as well as the motor cortex connectivity analysis are unlikely to be explained by any methodological differences or differences of non-neuronal origin.

## Discussion

This study supports the hypothesis that TRD is associated with reduced FC of neurocognitive networks in comparison to non-TRD patients and HC. Results showed decreased FC in TRD patients relative to both non-TRD patients and HC between (I) the anterior and posterior parts of the DMN; (II) the CCN and the posterior DMN, and (III) the motor cortex and superior temporal gyrus.

The anterior and posterior DMN subsystems have previously been found to show altered FC in depression, associated with rumination and overgeneralization of autobiographical memory (28). Furthermore, the connectivity between these two subsystems has been found to be reduced in depression (29). Our results indicate that such disturbed interaction between these DMN subsystems is also present in TRD. It should be noted that, besides showing dissociation between the DMN subsystems, these studies also found increased FC within these subsystems. We did not find any within-subsystem connectivity increases, which may be explained by the differences in applied methodology (ICA in the studies above versus seed-based analyses in our study). Furthermore, Greicius et al. found increased connectivity of the DMN with the subgenual ACC and thalamus (8). One explanation for the discrepancy with our results may be the inclusion of psychotic MDD patients in the study by Greicius et al., for example, previous research showed differences in subgenual ACC metabolism between psychotic and non-psychotic MDD subjects (45). Second, connectivity decreases between the CCN and DMN have also previously been demonstrated in MDD (12, 46), although increased connectivity has been found as well (9). As described by Hamilton et al., increased DMN dominance over the task positive network (TPN, which the CCN can be considered to be part of) is associated with maladaptive rumination in MDD (47). Decreased connectivity between the DMN and CCN in our TRD patients may related to DMN-TPN/CNN dominance and hence to depressive rumination. Third, we unexpectedly found decreased FC in TRD from the motor cortex seed region. Previous research using principal component analysis indicated a lack of segregation between regions involved in emotional, linguistic, DMN, and also motor functions in depression, which was thought to represent the interconnection of affective disturbance with experience, cognition, and behavior (48). Speculatively, our finding of more widespread decreased FC in TRD may also reflect such interconnection.

Surprisingly, the comparison between non-TRD patients and HC did not show any differences in FC in any of the three investigated networks. This was unexpected, since, as described above, several studies showed decreased FC within and between these neurocognitive networks in MDD (12, 29, 46). However, several MDD patients in these studies used antidepressant medication at time of scanning. Despite their treatment, those patients were still depressed, suggesting that they might have been treatment resistant to some degree. Given the results of decreased connectivity of TRD compared to non-TRD in our study, inclusion of (partially) treatment resistant MDD patients might have led to the connectivity decreases found in these studies. At the same time, this suggests that the relative lack of connectivity differences between our non-TRD and HC groups may be related to the non-medicated and non-resistance status of our non-TRD patients, which could be important to address in future studies.

Our results did not show differences in FC between the SN and both the CCN and DMN in TRD patients relative to non-TRD patients and HC. This suggests that the observed connectivity reductions that also extended to the motor network are nevertheless relatively specific and do not reflect a generalized reduction of FC in TRD. Another resting-state FC study did show decreased connectivity between the insula and both the ACC and precuneus in TRD and in non-TRD patients relative to HC, suggesting decreased connectivity between the SN and the DMN in both depression groups (32). However, in the direct comparison between TRD and non-TRD patients, the SN network was relatively spared in TRD. This suggests that any SN alterations in TRD are at least relatively modest, which may explain the negative findings in our study.

Taken together, our findings suggest that MDD-related dysfunction of neurocognitive networks may be particularly important in TRD. This raises the question whether TRD is a distinct MDD-subtype, characterized by specific DMN and CCN connectivity abnormalities. Another question is whether decreased FC is already present at the onset of the first depressive episode or evolves over time, with TRD being the end-stage of a progressively deteriorating course. If the latter is the case, decreased connectivity may be a neural marker for staging and profiling the severity of the disease as well as a target for secondary prevention (33, 37, 49). To answer these questions, longitudinal studies are needed with follow-up on treatment outcome, preferably accompanied by repeated scanning at different stages of the disease, starting during the first episode in medication-naïve patients. Moreover, further research is needed to investigate how decreased connectivity relates to depressive symptoms in TRD, e.g., by correlating connectivity with measures of rumination, autobiographical memory, or other clinical symptoms like anhedonia, cognitive impairments, and psychomotor retardation.

This study is one of the first to investigate resting-state FC in TRD and used a stringent correction for multiple comparisons to minimize the potential to report false positive results. However, this study also has particular methodological limitations that warrant further discussion. A first limitation is the presence of methodological differences in data acquisition between the TRD and non-TRD group (35 versus 40 slices; eyes open versus eyes closed), which may have influenced the comparison between these two groups. Additional control analyses, however, suggest that those differences unlikely explain the observed results: (I) comparison between the TRD group and those HC investigated with the same scanning protocol and instructions yielded a similar pattern of results as the comparison between the TRD and complete HC group; (II) connectivity from a white matter seed did not show any significant differences between TRD, non-TRD, and HC groups; (III) to account for differences in brain coverage between the two scanning protocols, we applied an inclusive mask that only included data present in all subjects; (IV) regarding the eyes open (TRD) versus the eyes closed (non-TRD) instructions, previous studies either found similar DMN connectivity maps (22, 50) or increased DMN connectivity when eyes where open compared to closed (51, 52). Thus, if this instruction would nevertheless have influenced the results, it is expected that this would have resulted in reduced rather than enhanced differences between the TRD and both the non-TRD and HC group (i.e., with eyes closed the connectivity in the TRD group would have been even lower).

A second methodological limitation is that we used a repetition time of 2300 ms. Although a shorter repetition time may be optimal for resting-state fMRI, this repetition time is comparable with many recent clinical resting-state fMRI studies (2200–2500 ms) (9, 26, 53). A third limitation concerns the difference in medication use: antidepressant medication was allowed in the TRD group, whereas the non-TRD group was medication-free at the time of scanning. Due to illness severity, TRD patients could not taper medication before scanning. Although statistical correction for medication use is often considered, this was not possible in our study, since the TRD group included only medicated patients, whereas the non-TRD group included only medication-free patients. As antidepressant medication has been found to increase FC between ACC and limbic areas in depressive patients and normalizes DMN dysfunction (54, 55), we assume that antidepressants in the present TRD group also may have partly normalized abnormal between network FC. Therefore, we expect that antidepressant use might only have reduced the differences between the TRD and both the non-TRD and HC group, which were still identified. Nevertheless, our assumption of normalizing effects of antidepressants is challenged by studies of prolonged antidepressant administration in HC, which appeared to decrease FC (56, 57). Therefore, no definite conclusions about the effects of medication in this study can be drawn, which warrants further investigation.

In conclusion, this study supports the hypothesis that severe TRD is characterized by a dysfunction of neurocognitive networks compared to non-TRD and HC, including decreased FC between the CCN and the DMN, as well as decreased connectivity between the anterior and posterior parts of the DMN. However, considering the present methodological limitations, our findings need replication by future research. Furthermore, longitudinal studies are needed to investigate how FC abnormalities in TRD evolve over time and to answer the question whether these abnormalities are a reflection of disease progression or initial abnormalities relative to non-TRD. Eventually, we may be able to map the risk of the development of TRD early, which might signal a “window of opportunity” for strategies to defer the fate of chronic suffering of TRD patients.

## Conflict of Interest Statement

The authors declare that the research was conducted in the absence of any commercial or financial relationships that could be construed as a potential conflict of interest.

## Supplementary Material

The Supplementary Material for this article can be found online at http://www.frontiersin.org/Journal/10.3389/fpsyt.2015.00028/abstract

## Footnotes

^1^http://www.fil.ion.ucl.ac.uk/spm/software/spm8/

^2^http://resting-fmri.sourceforge.net

^3^http://fmri.wfubmc.edu/cms/software#NS
