# Supplementary material for: Decreased Resting-State Connectivity between Neurocognitive Networks in Treatment Resistant Depression
Source: Front Psychiatry. 2015 Mar 2;6:28. doi: 10.3389/fpsyt.2015.00028 (PMC4345766; doi:10.3389/fpsyt.2015.00028)
Supplement: Supplementary file 1 [file Data_Sheet_1.DOC]

### Decreased resting-state connectivity between neurocognitive networks in treatment resistant depression compared to non-treatment resistant depression

**Supplemental information**

A

eFigure 1. Seed regions that were used to probe the different networks of interest. a) salience network (SN) with left (-32, 24, -6 mm) and right anterior insula (37, 25, -4 mm) [Sridharan, Levitin, and Menon 2008]. b) cognitive control network (CCN) with left and right dorsolateral prefrontal cortex (+/-36, 27, 29 mm) [Sheline et al. 2010]. c) posterior default mode network (DMN) with posterior cingulate cortex (-2, -51, 27 mm) [Uddin et al. 2009]. d) anterior default mode network (DMN) with medial prefrontal cortex (-1, 47, -4 mm) [Shulman et al. 1997] (all coordinates in MNI space).

**eTable 1. Functional connectivity analysis with bilateral motor cortex as seed region in comparison between TRD, non-TRD and healthy controls.**

| **Comparison** | **Brain region** | **MNI coordinates** | | | **Cluster Size** | **p*** |
| --- | --- | --- | --- | --- | --- | --- |
|  |  | **x** | **y** | **z** |  |  |
| **Seed region: Right motor cortex** | | | | | | |
| **F-test** | Right Superior temporal gyrus | 66 | 4 | 6 | 107 | 0.010 |
| Non-TRD > TRD | Right Superior temporal gyrus  PCC | 62  -22 | 4  -64 | 6  6 | 237  477 | 0.002  <0.001 |
| Non-TRD > HC | None |  |  |  |  |  |
| HC > TRD | Superior temporal gyrus | -66 | -4 | 10 | 151 | 0.018 |
| **Seed region: Left motor cortex** | | | | | | |
| **F-test** | Left Superior temporal gyrus | -66 | 0 | 6 | 153 | 0.003 |
| Non-TRD > TRD | Right Superior temporal gyrus  Left Inferior frontal Gyrus  PCC | 50  -58  -22 | -4  12  -68 | -10  22  -6 | 199  252  430 | 0.005  0.001  <0.001 |
| Non-TRD> HC | None |  |  |  |  |  |
| HC > TRD | Left superior temporal gyrus | -66 | 0 | 6 | 241 | 0.002 |

Abbreviations: HC, healthy controls; MDD, major depressive disorder (=TRD + non-TRD); TRD, therapy resistant depression; non-TRD, non-therapy resistant depression; MNI, Montreal Neurological Institute space coordinates.

**^*^**Corrected for multiple comparisons with family wise error correction (FWE) on cluster level.

Reference List

[1] Sheline, Y.I., Price, J.L., Yan, Z., and Mintun, M.A. (2010). Resting-state functional MRI in depression unmasks increased connectivity between networks via the dorsal nexus. *Proc Natl Acad Sci U.S.A* 107, 11020-11025.

[2] Shulman, G.L., Fiez, J.A., Corbetta, M., Buckner, R.L., Miezin, F.M., Raichle, M.E., and Petersen, S.E. (1997). Common Blood Flow Changes across Visual Tasks: II. Decreases in Cerebral Cortex. *J Cogn Neurosci.* 9, 648-663.

[3] Sridharan, D., Levitin, D.J., and Menon, V. (2008). A critical role for the right fronto-insular cortex in switching between central-executive and default-mode networks. *Proc.Natl.Acad Sci.U.S.A* 105, 12569-12574.

[4] Uddin, L.Q., Kelly, A.M., Biswal, B.B., Xavier, C.F., and Milham, M.P. (2009). Functional connectivity of default mode network components: correlation, anticorrelation, and causality. *Hum.Brain Mapp.* 30, 625-637.
